# A supervised machine-learning analysis of doxorubicin-loaded electrospun nanofibers and their anticancer activity capabilities

**DOI:** 10.3389/fbioe.2025.1493194

**Published:** 2025-03-11

**Authors:** Mohammadreza Rostami, Maliheh Gharibshahian, Mehrnaz Mostafavi, Ali Sufali, Mahsa Golmohammadi, Mohammad Reza Barati, Reza Maleki, Nima Beheshtizadeh

**Affiliations:** ^1^ Department of Nutrition, School of Allied Medical Sciences, Ahvaz Jundishapur University of Medical Sciences, Ahvaz, Iran; ^2^ Food Science and Nutrition group (FSAN), Universal Scientific Education and Research Network (USERN), Tehran, Iran; ^3^ Nervous System Stem Cells Research Center, Semnan University of Medical Sciences, Semnan, Iran; ^4^ Department of Tissue Engineering and Applied Cell Sciences, School of Medicine, Semnan University of Medical Sciences, Semnan, Iran; ^5^ Faculty of Allied Medicine, Shahid Beheshti University of Medical Sciences, Tehran, Iran; ^6^ Computational Biology and Chemistry Group (CBCG), Universal Scientific Education and Research Network (USERN), Tehran, Iran; ^7^ Department of Polymer Engineering and Color Technology, Amirkabir University of Technology, Tehran, Iran; ^8^ Department of Advanced Materials and New Technologies, Iranian Research Organization for Science and Technology (IROST), Tehran, Iran; ^9^ Department of Chemical Technologies, Iranian Research Organization for Science and Technology (IROST), Tehran, Iran; ^10^ Department of Tissue Engineering, Faculty of Advanced Medical Sciences, Tabriz University of Medical Sciences, Tabriz, Iran; ^11^ Regenerative Medicine group (REMED), Universal Scientific Education and Research Network (USERN), Tehran, Iran

**Keywords:** machine learning, anticancer activity, electrospun nanofibers, electrospinning, doxorubicin, artificial intelligence

## Abstract

Thanks to the diverse advantages of electrospun nanofibers, multiple drugs have been loaded in these nanoplatforms to be delivered healthily and effectively. Doxorubicin is a drug used in chemotherapy, and its various delivery and efficacy parameters encounter challenges, leading to the seeking of novel delivery methods. Researchers have conducted numerous laboratory investigations on the encapsulation of doxorubicin within nanofiber materials. This method encompasses various parameters for the production of fibers and drug loading, categorized into device-related, material-related, and study design parameters. This study employed a supervised machine-learning analysis to extract the influencing parameters of the input from quantitative data for doxorubicin-loaded electrospun nanofibers. The study also determined the significance coefficient of each parameter that influences the output results and identified the optimum points and intervals for each parameter. Our Support Vector Machine (SVM) analysis findings showed that doxorubicin-loaded electrospun nanofibers could be optimized through employing a machine learning-based investigation on the polymer solution parameters (such as density, solvent, electrical conductivity, and concentration of polymer), electrospinning parameters (such as voltage, flow rate, and distance between the needle tip and collector), and our study parameters, i.e., drug release and anticancer activity, which affect the properties of the drug-loaded nanofibers, such as the average diameter of fiber, anticancer activity, drug release percentage, and encapsulation efficiency. Our findings indicated the importance of factors like distance, polymer density, and polymer concentration, respectively, in optimizing the fabrication of drug-loaded electrospun nanofibers. The smallest diameter, highest encapsulation efficiency, highest drug release percentage, and highest anticancer activity are obtained at a molecular weight between 80 and 474 kDa and a doxorubicin concentration of at least 3.182 wt% with the polymer density in the range of 1.2–1.52 g/cm^3^, polymer concentration of 6.618–9 wt%, and dielectric constant of solvent more than 30. Also, the optimal distance of 14–15 cm, the flow rate of 3.5–5 mL/h, and the voltage in the range of 20–25 kV result in the highest release rate, the highest encapsulation efficiency, and the lowest average diameter for fibers. Therefore, to achieve optimal conditions, these values should be considered. These findings open up new roads for future design and production of drug-loaded polymeric nanofibers with desirable properties and performances by machine learning methods.

## 1 Introduction

Doxorubicin (DOX) is one of the most widely used chemotherapy drugs whose antitumor activity has been proven in treating a variety of cancers such as breast, lung, bladder, ovary, and stomach (Sohail et al., 2021). This drug is a class I anthracycline antibiotic with hydrophilic and fluorescent properties (Ansari et al., 2024). It acts on the S phase of the cell cycle and interferes with the structure and synthesis of DNA. DOX causes cell death by interfering with nucleotides, inhibiting topoisomerase II, and producing oxygen-free radicals (Pal, 2024). However, this drug has a short half-life, and a high dose is needed to achieve the desired therapeutic index, which leads to toxicity and various side effects on normal cells (Rivankar, 2014). For example, intravenous injection of high doses causes alopecia, vomiting, cell suppression, myelotoxic poisoning, gastrointestinal toxicity, cardiac toxicity, and various arrhythmias (Schrijvers et al., 2023). These issues highlight the necessity of developing a suitable carrier system to reduce side effects, reduce cost, and prevent local cancer recurrence.

Over the past few years, researchers have developed various carrier systems to improve the effectiveness of the DOX for chemotherapy by lowering the required dose and increasing its therapeutic outcomes. Among these systems, we can mention liposomes (Pal et al., 2023), dendrimers (Singh and Kesharwani, 2021), micelles (Almajidi et al., 2023), nanoparticles (Harris et al., 2022), and nanofibers (Bahmani et al., 2024). Nanofibers are promising carriers for DOX due to their high surface-to-volume ratio, porosity between fibers, large specific surface area, suitable mass transfer, and the ability to tailor encapsulation efficiency and drug release profile by modifying the features of the nanofibers, such as morphology, fiber diameter, and composition, and adding different functional groups to the surface of nanofibers (Mozaffari et al., 2022).

Electrospinning is a simple and widely used method to scale up nanofiber fabrication from all kinds of polymers, ceramics, composites, and semiconductors (Venmathi Maran et al., 2024). This method produces fibers with diameters ranging from a few nanometers to a few micrometers by applying a strong electric field to the polymer solution. The electric field evenly distributes the induced charges on the surface of the polymer drop. The electrospinning process involves inducing a uniform distribution of charges on the polymer drop at the tip of the needle, initiating the polymer spinning through the generation of a strong enough electric force (applied voltage), overcoming the surface tension of the polymer droplet, collecting the charged polymer nanofibers on the grounded metal plate or collector, and finally obtaining a polymer mat after solvent evaporation (Keirouz et al., 2023).

**FIGURE 1:**
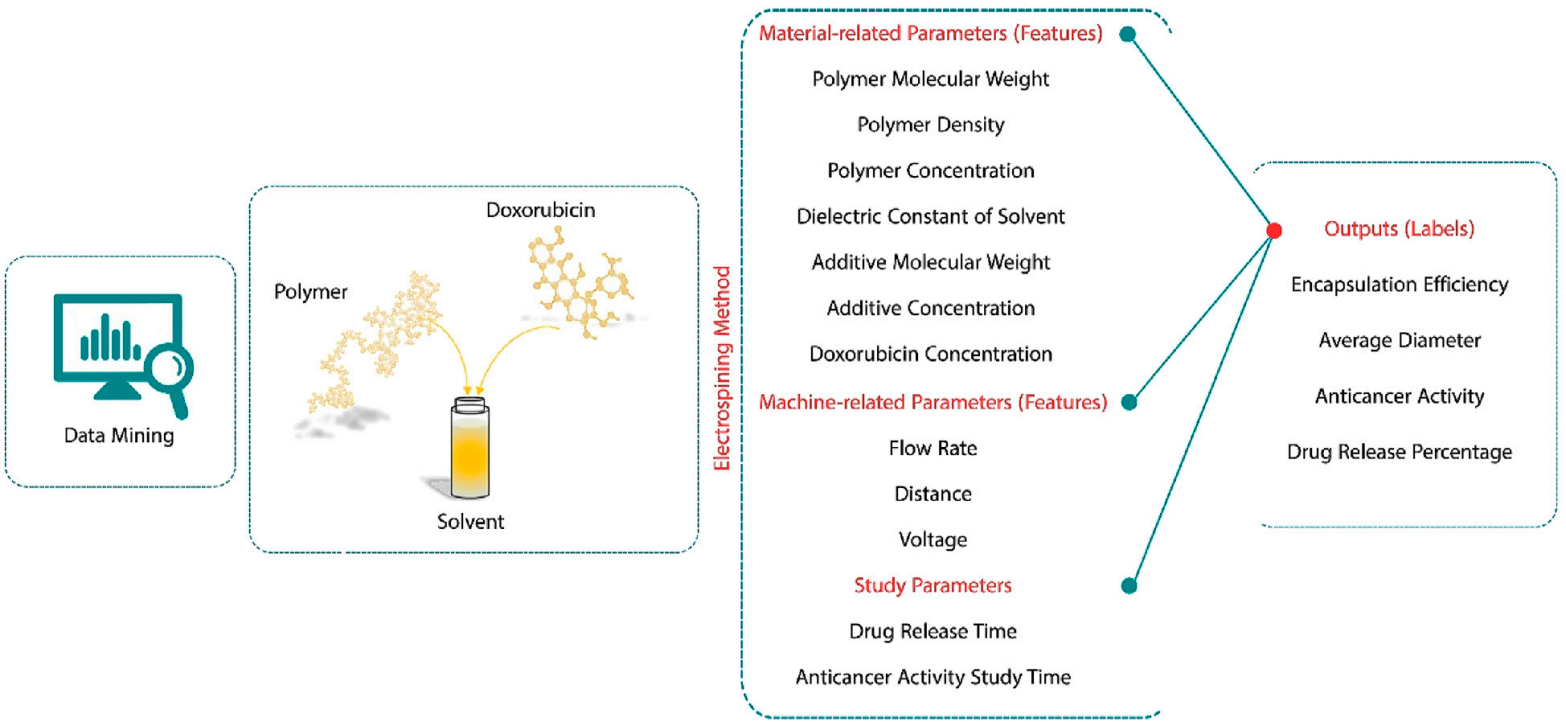
A schematic diagram illustrates that electrospinning is a multi-parameter method in which various parameters, including polymer solution parameters (such as molecular weight, density, dielectric constant of solvent, additive molecular weight, additive concentration, and drug concentration), machine parameters (such as voltage, flow rate, and distance between needle tip and collector), and study parameters such as drug release and anticancer activity study times, affect the properties of the resultant nanofibers, such as the diameter of the fibers, the amount of drug loading and release, encapsulation efficiency, and anticancer activity.

Drug-carrying nanofibers offer the advantage of delivering multiple drugs, along with high encapsulation efficiency and affordability. Electrospinning can generate fibers with a core-shell structure to incorporate different drug release profiles. However, electrospinning is a multi-parameter method in which various parameters, including polymer solution parameters (such as molecular weight, density, solvent, electrical conductivity, and concentration), machine settings (such as voltage, flow rate, and distance between needle tip and collector), and environmental conditions (such as temperature and moisture), affect the characteristics of the resultant nanofibers, like the diameter of the fibers, the amount of drug loading and release, the mechanical properties of nanofibers, and the therapeutic effects of the drug (Figure 1). On the other hand, it is impossible to control and check all these parameters simultaneously in *vitro* and *in vivo* evaluations. *In silico* computational and optimization algorithms could be a useful facility in predicting and controlling the mentioned parameters. Artificial intelligence (AI) technology can aid in assessing medicine and drug release systems. Although some environmental parameters, such as temperature, humidity, pressure, and solvent boiling point can also affect the procedure, they have been ignored in most studies, and utilizing them was unavailable.

Integrating AI and machine learning (ML) approaches with nanomedicine and tissue engineering technologies leads to the design and development of smart drug release systems with rational design, high efficiency, and predefined functions (Lin et al., 2022). This advancement accelerates the transfer of drug-carrier systems from research settings to their practical use in clinical settings. The rapid growth of computing power, a large volume of data, and different data analysis algorithms have made it possible for AI to assess and predict all the parameters mentioned above in DOX carriers based on existing research findings in the literature (Lin et al., 2022; Serov and Vinogradov, 2022).

Numerous studies have explored the application of machine learning computations in electrospinning from different perspectives (Sukpancharoen et al., 2024; Subeshan et al., 2024). Toscano et al. (2020) carefully examined the electrospinning of polyacrylonitrile (PAN) at a concentration of 10% using a combination of experimental investigation and data-driven modeling. They reported subjecting the acquired data to three machine learning techniques: Lasso, random forest (RF), and support vector machine (SVM). According to their study, the RF model demonstrates a high level of predictability, with an average relative error of around 15% when making predictions on testing data over 100 replications (Toscano et al., 2020).

Pervez et al. (2023a) used Taguchi’s statistical orthogonal design to construct a model known as locally weighted kernel partial least squares regression (LW-KPLSR) for predicting the diameter of the electrospun nanofiber membrane made from chitosan. Based on their study, through employing the LW-KPLSR model, the coefficient of determination (R^2^) reached 0.9996, which is an exceptional accuracy value in ML modeling techniques.

Although other researchers have made efforts to explore the novelties of ML application in solving electrospinning challenges and predicting the resulting outcomes (Pervez et al., 2023b; Sarma et al., 2022; Shin et al., 2021; Wang et al., 2020), the topic is still a broad and underexplored area, and studies have not comprehensively covered the key aspects of this field. ML techniques can successfully train on drug-loaded nanofibers, specifically encapsulated via electrospinning techniques, to predict the system and its outputs (Khedri et al., 2022). Therefore, there is an urgent need to comprehensively explore the potential of ML in enhancing both the efficiency and effectiveness of electrospinning, especially in drug delivery systems that employ electrospun fibers. Hence, this study focuses on developing a new supervised machine-learning analysis of doxorubicin-loaded electrospun nanofibers. The primary objective is to determine the optimal parameters for the electrospinning of drug-loaded nanofibers across different materials to achieve the best loading efficiency, a high drug release profile, and enhanced therapeutic efficacy.

## 2 Materials and methods

Multiple previously published *in vitro* and *in vivo* evaluations on DOX-loaded electrospun nanofibers were considered, and their corresponding data were collected by the authors. For data analysis and the machine learning approach, four sequential steps were taken as follows: (1) data preprocessing (data cleaning), (2) feature importance, (3) model training and evaluation, and (4) model optimization. The applied dataset consisted of 12 features and 4 labels. The features were divided into two main categories as fixed and additional features, in which the first category included two subgroup features as *(i)* inputs related to the machine conditions (flow rate (mL/h), distance (cm), and voltage (kV)); and *(ii)* materials associated parameters, which consisted of polymer molecular weight (Mw, kDa), polymer density (g/cm^3^), additive molecular weight (Mw, kDa), additive concentration (wt.%), DOX concentration (wt.%), dielectric constant of solvent, and polymer concentration (wt.%). The latter feature category included drug release time (day) and anticancer activity study time. Table 1 summarizes the details of all feature categories. Moreover, in this study, average diameter (nm), encapsulation efficiency, drug release percentage, and anticancer activity were considered as outputs or labels.

**TABLE 1:** Input features of the current study.

	Inputs	
Fixed features	Flow rate (mL/h)	(Machine conditions)
	Distance (cm)	
	Voltage (kV)	
	Polymer molecular weight (kDa)	(Materials associated parameters)
	Polymer density (g/cm^3^)	
	Additive molecular weight (kDa)	
	Additive concentration (wt.%)	
	Doxorubicin concentration (wt.%)	
	Dielectric constant of solvent	
	Polymer concentration (wt.%)	
Additional features	Drug release time (day)	
	Anticancer activity study time	

### 2.1 Data cleaning

In the first step of data cleaning, the Box-Cox transformation was applied to transform each of the dataset columns (both features and labels) to achieve a dataset with the normal distribution.

### 2.2 Feature importance

The next step involved removing 126 missing values from the dataset. The imputation process was performed using the fancyimpute package and Soft-Impute for imputing missing values. The missing features were approximated, in which each feature or label was scaled between 0 and 1 via min-max scaling to guarantee comparability and consistency in the dataset. This scaling for a feature or label X of a data sample is shown in Equation 1:

$$X_{Scaled}=\frac{X-X_{\mathrm{Min}}}{X_{\mathrm{Max}}-X_{\mathrm{Min}}}$$ (1)

Where X is the feature or label that is desired to be labeled, and X_Min_, X_Max_, and X_Scaled_ are the minimum, maximum, and scaled values of the desired feature or label, respectively. The dependence of each label on another was investigated through the feature importance step prior to the modeling step. For measuring the feature importance of each label, Spearman’s rank correlation coefficient was calculated between one specific output and various inputs for finding the strength of a relationship between a pair of features or labels, which ranges from 1 to −1.

Although there are various correlation coefficients, in this study, Spearman’s correlation coefficient was applied because of its promising advantages, which include simplicity, non-linearity support, and computational efficiency. Equations 2 and 3 can calculate the Spearman’s correlation coefficient for a pair of features and labels X and Y. This coefficient helps to understand the interdependencies among variables in the dataset.

$$r_{s}=1-\frac{6\sum d_{i}^{2}}{n\left(n^{2}-1\right)}$$ (2)

$$d_{i}=R\left(X_{i}\right)-R\left(Y_{i}\right)$$ (3)

Where $R\left(X_{i}\right)$ and $R\left(Y_{i}\right)$ are rank values of the *ith* data correspond to X (feature) and Y (label), respectively. It should be highlighted that the time considered for evaluating the drug release % is effective in the acquired values for drug release percentage. Also, investing time in researching the anticancer activity is efficient in anticancer activity data outcomes. This information guides the selection of training datasets for the development of ML models. Finally, Table 2 summarizes the import of four groups of datasets for training the ML model.

**TABLE 2:** Used input features for each label.

Label (output)	Used features	Total features
Average diameter	10 fixed features	10
Encapsulation efficiency	10 fixed features	10
Anticancer activity	10 fixed features +1 additional feature (anticancer activity study time)	11
Drug release percentage	10 fixed features +1 additional feature (drug release time)	11

### 2.3 Model training and evaluation

The next step involved training and testing the SVM model. The SVM model is designed for regression and prediction of continuous values and seeks to find a function to accurately predict complex and nonlinear data by maintaining the maximum margin between the data points and the prediction function and keeping the prediction errors within a certain range (ε). However, careful parameter tuning and proper kernel selection are necessary for optimal performance. Parameters and values for developing the SVM model for each label are outlined in Table 3.

**TABLE 3:** Parameters of trained SVM model.

Labels	Gamma	C	Epsilon
Average diameter	41.50438	32.19417	6.44520 × 10^−5^
Encapsulation efficiency	1.16007	30.52169	1.23861 × 10^−3^
Drug release percentage	3.49121	12.33292	1.60926 × 10^−5^
Anticancer activity	0.85080	41.04649	0.08052

The applied dataset was divided into two sections: a training set (80% of the whole dataset) and a testing set (20% of the whole dataset). For the evaluation of the model’s performance, three evaluation metrics, including Mean Absolute Error (MAE) (Equation 4), Mean Squared Error (MSE) (Equation 5), and Root Mean Squared Error (RMSE) (Equation 6), were tested and compared (Hyndman and Koehler, 2006). $R\left(X_{i}\right)$ and $R\left(Y_{i}\right)$ are rank values of the *ith* data correspond to X (feature) and Y (label), respectively.

$$\text{MAE}=\frac{1}{n}\;\sum_{i=1}^{n}\left|Y_{i}-{f\left(X\right)}_{i}\right|$$ (4)

$$\text{MSE}=\frac{1}{n}\;\sum_{i=1}^{n}{\left(Y_{i}-f\left(X_{i}\right)\right)}^{2}$$ (5)

$$\text{RMSE}=\sqrt{\frac{1}{n}\sum_{i=1}^{n}{\left(Y_{i}-f\left(X_{i}\right)\right)}^{2}\;}$$ (6)

### 2.4 Model optimization

The optimization process was performed via the Particle Swarm Optimization (PSO) algorithm to fine-tune three key control optimization parameters, such as C1 (cognitive coefficient), C2 (social coefficient), and Omega (inertia weight). C1 determines how much a particle should be influenced by its personal best position, while C2 controls the influence of the global best position found by the swarm. Omega controls the particle’s momentum, affecting how much it maintains its previous velocity. Through trial and error optimization, we determined the optimal values to be C1 = 0.4862, C2 = 2.5067, and Omega = −0.2887 (Pedersen, 2010). These values balance the algorithm’s exploration and exploitation capabilities: the relatively low C1 reduces individual particle bias, the higher C2 encourages convergence toward the global best solution, and the negative Omega helps prevent premature convergence by allowing particles to change direction more readily (Pereira, 2011).

## 3 Results and discussion

### 3.1 Evaluation of model accuracies

The dataset of this study contained 12 inputs (features) and four outputs (labels), which were tested through the SVM model. As mentioned previously, three evaluation indices, namely, MAE, MSE, and RMSE, measured the accuracy of the applied model. The final results are summarized in Table 4, demonstrating the capability and precision of the predictive model in elucidating the relationships between the input variable and output labels. Also, scatter-plots are presented in Supplementary Figures S1–S4.

**TABLE 4:** Evaluation indices of the trained model performances.

Label	MAE	MSE	RMSE
Average Diameter	43.74205	8361.02123	91.43862
Encapsulation Efficiency	2.35201	117.63967	10.84618
Drug Release Percentage	3.21023	47.71458	6.90757
Anticancer Activity	13.14296	99.67285	9.98362

MAE is a simple interpretation of the average magnitude of errors in a set of forecasts (regardless of their magnitude and direction) that uses the same units as the output variable to express the average error. Since MAE uses the same penalty for large and small errors, it is not desirable in applications where larger errors are more important (Hyndman and Koehler, 2006). MSE can give greater weight and sensitivity to larger errors than outliers by measuring the mean squared errors (the mean squared difference between the estimated values and the true value) and squaring the differences. However, this squaring of errors causes the adverse effect of several large errors on the overall metric and does not accurately reflect the performance of the model (Hastie et al., 2009).

RMSE, by expressing the root mean squared errors in the same units as the output variable, is known as a measure of the degree of dispersion of the residuals and provides a satisfactory degree of data division ratio. A smaller RMSE indicates better accuracy, but the high impact of outliers in it leads to a lack of a complete picture of the model’s performance (Pervez et al., 2023b). Utilizing these criteria, it is possible to obtain a comprehensive assessment of the forecasting performance of SVM models and determine the error distribution, overall accuracy, and sensitivity of this model to outliers.

### 3.2 Feature importance

**FIGURE 2:**
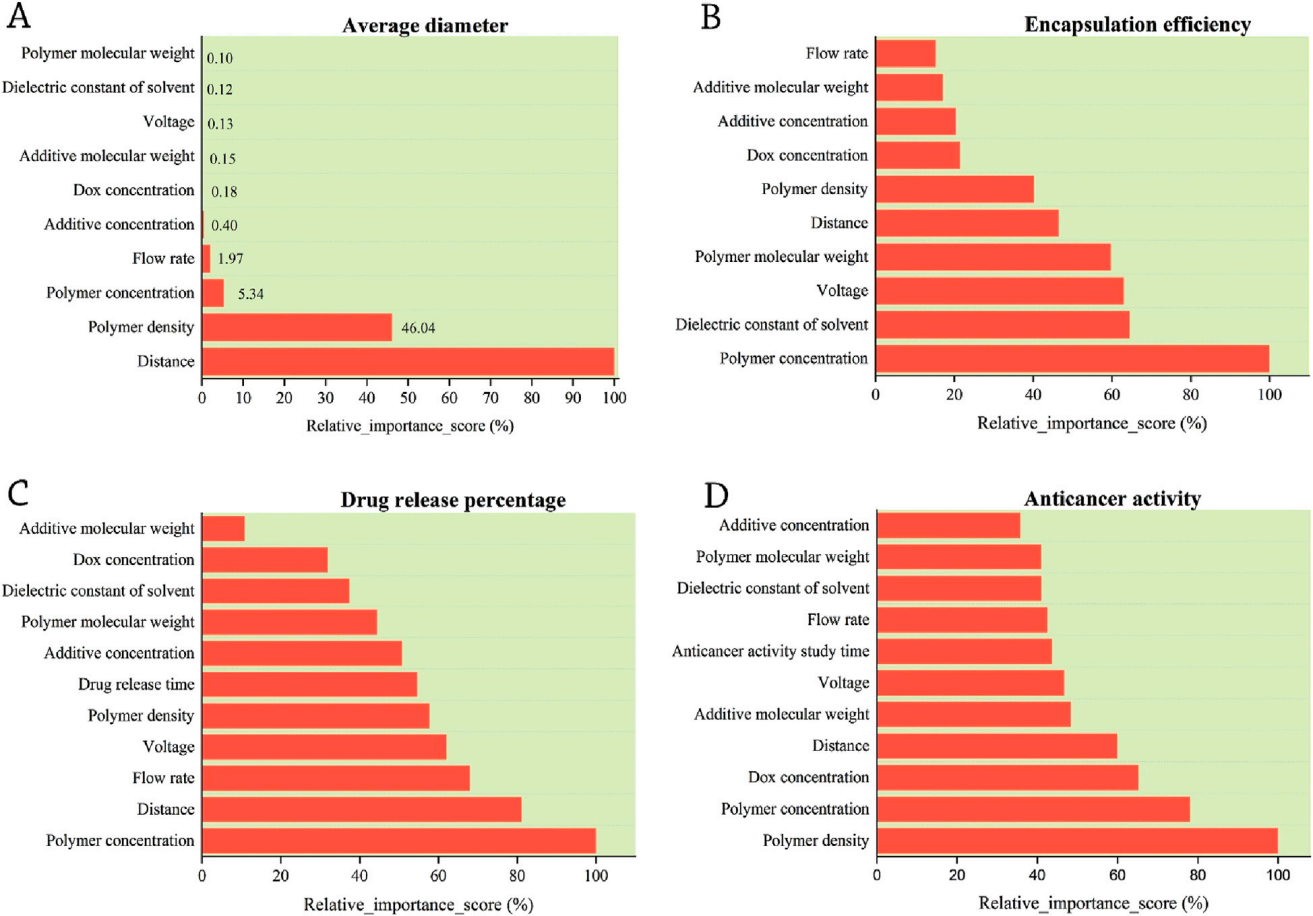
Relative feature importance score (%) of **(A)** average diameter, **(B)** encapsulation efficiency, **(C)** drug release percentage, and **(D)** anticancer activity. A highly important feature for the average diameter is distance, which is a machine condition feature. However, three other labels, including encapsulation efficiency, drug release percentage, and anticancer activity, are highly dependent on the material’s associated parameters and features. For encapsulation efficiency and drug release percentage, the most important parameter is polymer concentration, and anticancer activity is dependent on the polymer density more than other parameters.

Feature importance analysis resulted in measuring the importance score and relative importance score (%). The relative importance scores (%) for each label are depicted in Figure 2. As shown, among the four investigated labels in this study, the average diameter of nanofibers is strongly influenced by the distance, which is a machine-condition feature. However, three other labels, including encapsulation efficiency, drug release percentage, and anticancer activity, are highly influenced by the features of the materials used for the fabrication of nanofibers. For encapsulation efficiency and drug release percentage, the polymer concentration plays a significant role, and anticancer activity is dependent on the polymer density.

Figure 2 demonstrated that the factors that significantly affect the average diameter, such as distance and polymer density, have a more direct influence on the physical properties and structure of the polymer layer. Parameters like polymer concentration and flow rate might have a smaller, more subtle effect because their impact is only noticeable under certain conditions or within a specific range. The other parameters might be optimized or controlled in such a way that they do not significantly alter the average diameter, thus leading to their minimal effect in our model.

### 3.3 Model optimization

As previously mentioned, PSO is used for hyperparameter tuning. The results of the feature optimization are summarized in Table 5. This table shows the optimization points of each feature for reaching the minimum average diameter and the maximum encapsulation efficiency, drug release percentage, and anticancer activity. For instance, for obtaining the minimum average diameter, the maximum encapsulation efficiency, the drug release percentage, and the anticancer activity, polymer density should be 1.5299, 1.1960, 1.1983, and 1.5207, respectively. The optimization results are depicted in Figure 2, focusing on the three features with the highest relative importance score according to their investigated labels, as tabulated in Table 6.

**TABLE 5:** Features optimization points for each label.

Label Features	The minimum average diameter (nm)	The maximum encapsulation efficiency	The maximum drug release percentage	The maximum anticancer Activity
Polymer Molecular Weight (kDa)	80.8597	474.8010	318.1338	262.0249
Polymer Density	1.5299	1.1960	1.1983	1.5207
Additive Molecular Weight (kDa)	493.0598	188.3852	250.7143	83.3650
Additive Concentration (wt.%)	0.0848	22.3219	2.5661	23.2383
Dox Concentration (wt.%)	1.0044	9.6091	2.0584	3.1828
Dielectric Constant of Solvent	14.8257	65.1107	5.3971	25.8070
Polymer Concentration (wt.%)	0.2593	7.1982	6.6184	0.1391
Flow Rate (mL/h)	0.8129	15.0100	19.6483	6.2994
Distance (cm)	15.0380	13.0735	14.1093	19.5443
Voltage (kV)	19.9763	31.2323	46.1441	26.7860
Drug release time (hours)	__	__	0.2208	__
Anticancer activity study time (day)	__	__	__	15.4382

**TABLE 6:** Three features with the highest importance (for each label).

Label	Related most importance (for each label)
Average diameter	Distance Polymer density Polymer concentration
Encapsulation efficiency	Polymer concentration Dielectric constant of solvent Voltage
Drug release percentage	Polymer concentration Distance Flow rate
Anticancer activity	Polymer density Polymer concentration Dox concentration

As previously mentioned, electrospinning uses electrohydrodynamic processes to produce nano- and micro-scale fibers by placing a drop or polymer solution, which is placed under a high-voltage electric field. This method utilizes a device that includes parts of a feeding pump, a voltage source, and a material collection part. The feeding pump features a needle that receives an electric field. A Taylor cone of polymer solution forms at the end of the needle due to the interplay of the material flow from the feeding pump and the application of voltage (Keirouz et al., 2023). Next, the polymer solution is thrown towards the collector plate, and then the solvent in the solution evaporates, and dried and solid fibrous networks are formed.

Multiple applications for nano/micromaterials produced by this method are considered, from electronics and batteries to pharmaceuticals, food packaging, and medical applications, with a focus on tissue engineering, drug delivery, and nanoencapsulation. In medical applications, the produced nanofibers should meet particular characteristics, including size, surface-to-volume ratio, and porosity, which are evaluated experimentally to ensure the proper functionality of nanomaterials for target applications (Khedri et al., 2022). This work analyzes the DOX encapsulation in electrospun nanofiber mats using ML and carefully explores the optimal points for each of these parameters.

### 3.4 Average diameters of nanofibers

According to the analyses performed on the gathered data, among machine-related parameters like voltage, flow rate, and the distance between the needle and the collector, and material-related parameters like polymer concentration, polymer molecular weight, and DOX concentration, the most significant effective parameter on the size and diameter of the fibers is dedicated to the distance between the needle and the collector (Figure 2A). This coefficient of influence is based on the output obtained from ML training.

It should be noted that all device parameters and polymer solution characteristics are influential and important in the production and preparation of fibers. All these parameters are interdependent, such that without optimizing the concentration of the polymer solution, fibers cannot be produced at any distance. Furthermore, it can be emphasized that by altering the distance and operational conditions, more uniform and desirable nanofibers can be obtained. Hence, among the device parameters, the distance parameter has shown the greatest influence on the diameter of nanofibers.

**FIGURE 3:**
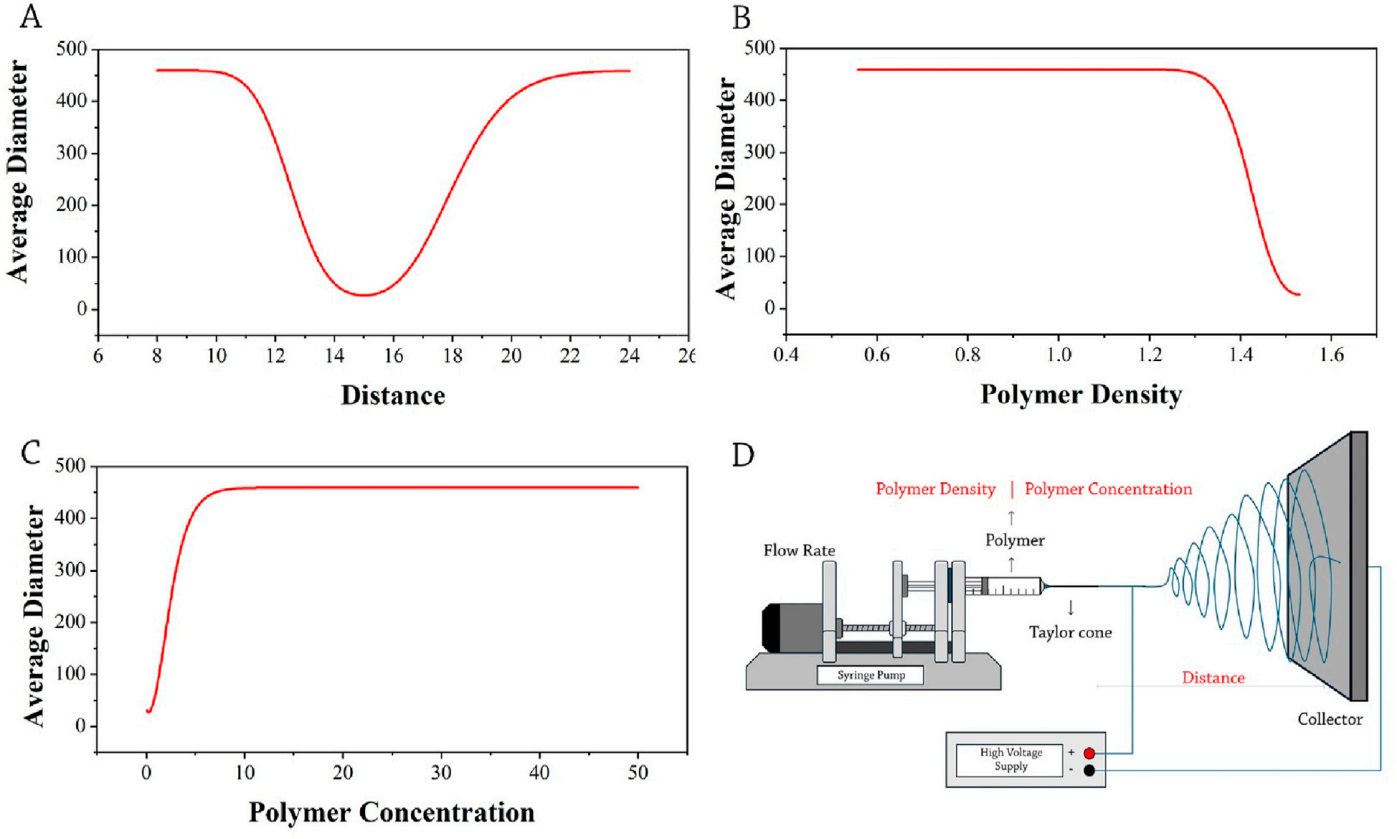
Optimization trends of average diameter: **(A)** distance, **(B)** polymer density, **(C)** polymer concentration, **(D)** schematic presentation of selected parameters (red-colored) influencing the average diameter of drug-loaded electrospun nanofibers.

Studies have shown that most previous works use a distance of 8–26 cm between the tip of the needle and the collector. Studies have shown that fibers with larger diameters are produced at shorter distances (Haider et al., 2018; Wen et al., 2021). It can be seen that by increasing the distance from 8 cm to 15 cm, the average diameter of the produced nanofibers decreases, and at 15 cm, the lowest diameter of nanofibers has been reported. The adjustment of the needle-to-collector distance is a parameter that largely depends on the properties of the polymer solution and the applied voltage of the device. As we move away from this area toward either end of the range, the production of fibers and their uniformity are expected to decrease (Figure 3A).

Previously, the ability of other machine learning models to estimate the diameter of electrospun fibers has been proven. Pervez et al. (2023b) developed a new LW-KPLSR model integrated with response surface methodology to predict nanofiber membrane diameter. Using electrospinning process data from three case studies, they investigated various parameters such as voltage, flow rate, polymer solution concentration, and tip-to-needle distance. Their results showed that the LW-KPLSR model performed better than other used models such as principal component regression (PCR), fuzzy, partial least square regression (PLSR), locally weighted partial least squares regression (LW-PLSR), and least square support vector regression model (LSSVR) models, which was proved by lower values of RMSE and MAE as well as high R^2^ (up to 0.9989). They showed that this model can help quickly optimize the electrospinning process and achieve the desired membrane diameter.

The polymer density, a crucial parameter affecting the average diameter of nanofibers, demonstrated a substantial influence, accounting for 46% of the variations in the average fiber diameter (Figure 2A). After comprehensive exploration in the literature, it is notable to report that the density of the polymers used in these studies varied between 0.5 and 1.6 g/cm^3^. Research findings indicate that by increasing the density of polymers from 0.5 to 1.2 g/cm^3^, the average diameter of fibers does not change; however, beyond the density of 1.3 g/cm^3^, the average diameter of the fiber decreases. A higher density of 1.529 g/cm^3^ creates the smallest diameter of nanofibers, suggesting an optimal density (Figure 3B). Polymer density influences parameters such as viscosity, surface tension, adhesion, and chain entanglement in the polymer solution. Probably, with the increase in the density of the polymer, the amount of chain entanglement in the polymer solution increases. This means that molecules and polymer chains stretch continuously.

Polymer concentration is another parameter that affects the ability to produce fibers from the polymer solution and, in turn, their shape and size (Angel et al., 2020). The data extracted from the previous studies shows the polymer concentration mostly varies from 1 to 50 wt.% in the polymer solution, and that the size of fibers increases as polymer concentration increases. With the increase in the viscosity of the solution used in the electrospinning process, the formation of larger cones at the tip of the needle leads to an increase in the diameter of the nanofibers (Khedri et al., 2022).

When viscosity increases, it affects how the material flows from the tip of the needle, resulting in the nanofibers not being able to stretch effectively, which in turn increases their diameter. Additionally, at the tip of the needle, due to increased viscosity, a larger Taylor cone is formed, causing the liquid to exit the needle more slowly. This can lead to the ejection of larger droplets of material, meaning that instead of forming a thin and continuous jet, larger masses of material are released from the needle. Ultimately, these factors work together to result in an increased diameter of the nanofibers. However, at concentrations beyond 9 wt.%, the average diameter of the fibers remains constant (Figure 3C).

Similar to the polymer density, the concentration of the polymer solution, affects the characteristics of viscosity, surface tension, adhesion, and chain entanglement (Ogazi and Osifo, 2023). Sarma et al. (2022) used black-box models and a model-agnostic interpretable game theory approach as an efficient approach to determine the optimal conditions for the electrospinning of polyvinylidene fluoride (PVDF) and the relationships between variables. Their results showed that PVDF fiber diameter is affected by various parameters such as polymer concentration, relative energy difference, and feed, which is in agreement with our results. Typically, a concentration below 9 wt.% could be regarded as a favorable concentration, efficient fiber production, and adaptable for multiple practical applications. A schematic presentation of selected parameters (red-colored) influencing the average diameter of the fiber is shown in Figure 3D.

Based on the analysis conducted, it is observed that nanofibers with a uniform structure, measuring below the average diameter of 500 nm, can be produced at concentrations below 10 wt.%. In fact, a concentration of 9 wt.% can be identified as the optimal concentration for the production of nanofibers under 500 nm. Studies focusing on drug incorporation into fibers have identified the average diameter, shape, porosity, and uniformity of fibers as essential factors for their proper practical applications, particularly in drug delivery applications where consistent homogeneity and small fiber sizes are key considerations (Abdulhussain et al., 2023).

### 3.5 Encapsulation efficiency

The utilization of electrospun nanofibers is highly valued for their notable capacity to efficiently load bioactive substances. These nanofibers are favored due to their ease of operation, cost-effectiveness, and high encapsulation efficiency, which are vital for efficient drug delivery applications like DOX loading into the fibers. The success of encapsulation efficiency in nanofiber materials can be controlled by several factors, including a high surface-to-volume ratio, significant porosity, and effective trapping facilitated by polymer chains.

In the analyses conducted on the impact ratio of the input parameters on the encapsulation efficiency, it has been ascertained that the parameter of polymer concentration holds significant importance, scoring 100%. The effectiveness coefficients for the parameters of solvent properties, voltage, polymer molecular weight, distance, and polymer density are 65%, 63%, 60%, 50%, and 40%, respectively (Figure 2B). These results emphasize the significance of managing different input parameters to maximize the encapsulation efficiency of bioactive compounds in nanofibers. This graph showing the influence coefficients of the input parameters and how they affect the amount of DOX encapsulation makes it clear that all of the parameters have an influence coefficient higher than 15%.

The efficacy of encapsulation is contingent upon the generation of appropriate and high-quality fibers, with all parameters playing a significant role in attaining this objective. Based on the optimal parameters, the encapsulation efficiency is significantly influenced by the polymer concentration.

**FIGURE 4:**
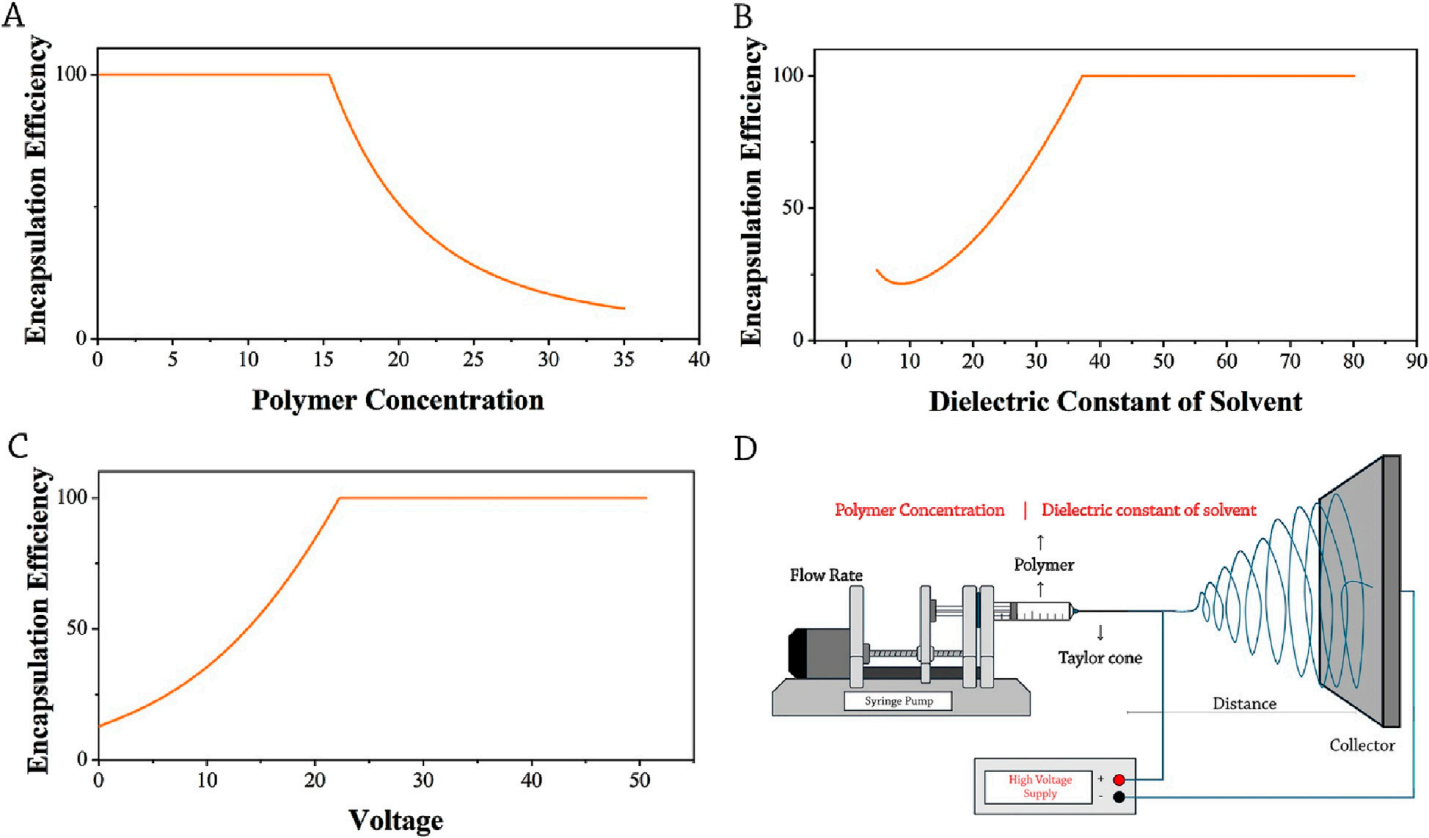
Optimization trends of encapsulation efficiency: **(A)** polymer concentration, **(B)** dielectric constant of solvent, **(C)** voltage, and **(D)** schematic presentation of selected parameters (red-colored) influencing the encapsulation efficiency of drug-loaded electrospun nanofibers.

Figure 4A shows that concentrations ranging from 0.1% to 16% have the highest encapsulation efficiency. When the concentration of polymers exceeds 20%, there is a reduction in drug loading. Such reduction in drug loading is likely attributed to a decline in the fiber production capability at higher polymer concentrations. Our data analysis demonstrated that there is a higher frequency of data falling within the range of 1%–16%.

Furthermore, as the range extends towards its upper limit, there is a noticeable decrease in loading efficiency. Yu et al. (2015) used the incorporation of DOX-carrying carbon nanotubes (CNTs) (DOX@CNTs) into electrospun poly (lactic-co-glycolic acid) (PLGA) nanofibers as a substrate for cancer therapy. Their results showed that the nanofibers had a smooth design and a uniform distribution of nanoparticles, and the PLGA/DOX@CNTs platform could effectively inhibit HeLa cell viability *in vitro*. The DOX was released from the composite nanofibers in a sustained and prolonged manner, and as a result, a significant anticancer effect was obtained in *in vitro* conditions. 20% polymer concentration leads to 81.50% encapsulation, and the change in the amount of CNTs does not change this amount, which indicates the importance of polymer concentration in drug encapsulation, which is in agreement with our results.

The dielectric constant is another significant factor that contributes to enhancing the encapsulation efficiency, demonstrating an influential value of 64% to enhance the encapsulation of DOX in the solvent (Figure 2B). It was found that changing the dielectric constant of the solvent from 1 to 40 results in a higher level of encapsulation efficiency; beyond the dielectric constant of 40, there is no further increase in the encapsulation efficiency. Hence, to obtain a higher encapsulation efficiency, a minimum value of solvent dielectric constant should be at least 40 (Figure 4B).

The fabrication process of electrospun fibers widely recognizes the voltage parameter as a highly influential factor. There is a consistent increase in encapsulation efficiency within the voltage range of 10–23 kV. It is important to acknowledge that the range commonly employed in electrospun fiber production research is typically between 10 and 35 kV (Figure 4C). There is a potential correlation between the voltage parameter and the dielectric constant in enhancing the efficacy of the drug, as both are associated with the characteristic of electric charge conduction. Liu et al. (2013) used Poly-L-lactic acid (PLLA) (6 wt.%) fibers loaded with DOX for local chemotherapy against secondary hepatocellular carcinoma (SHCC). The voltage of 1.9 kV and the dielectric constant of 11.22 led to the loading of only 6 wt.% of the drug in these nanofibers and the death of 90% of the tumor cells, which could be a proper confirmation for the parameters optimization via our ML model performed in this study. A schematic presentation of selected parameters (red-colored) influencing the encapsulation efficiency is shown in Figure 4D.

### 3.6 Drug release percentage

**FIGURE 5:**
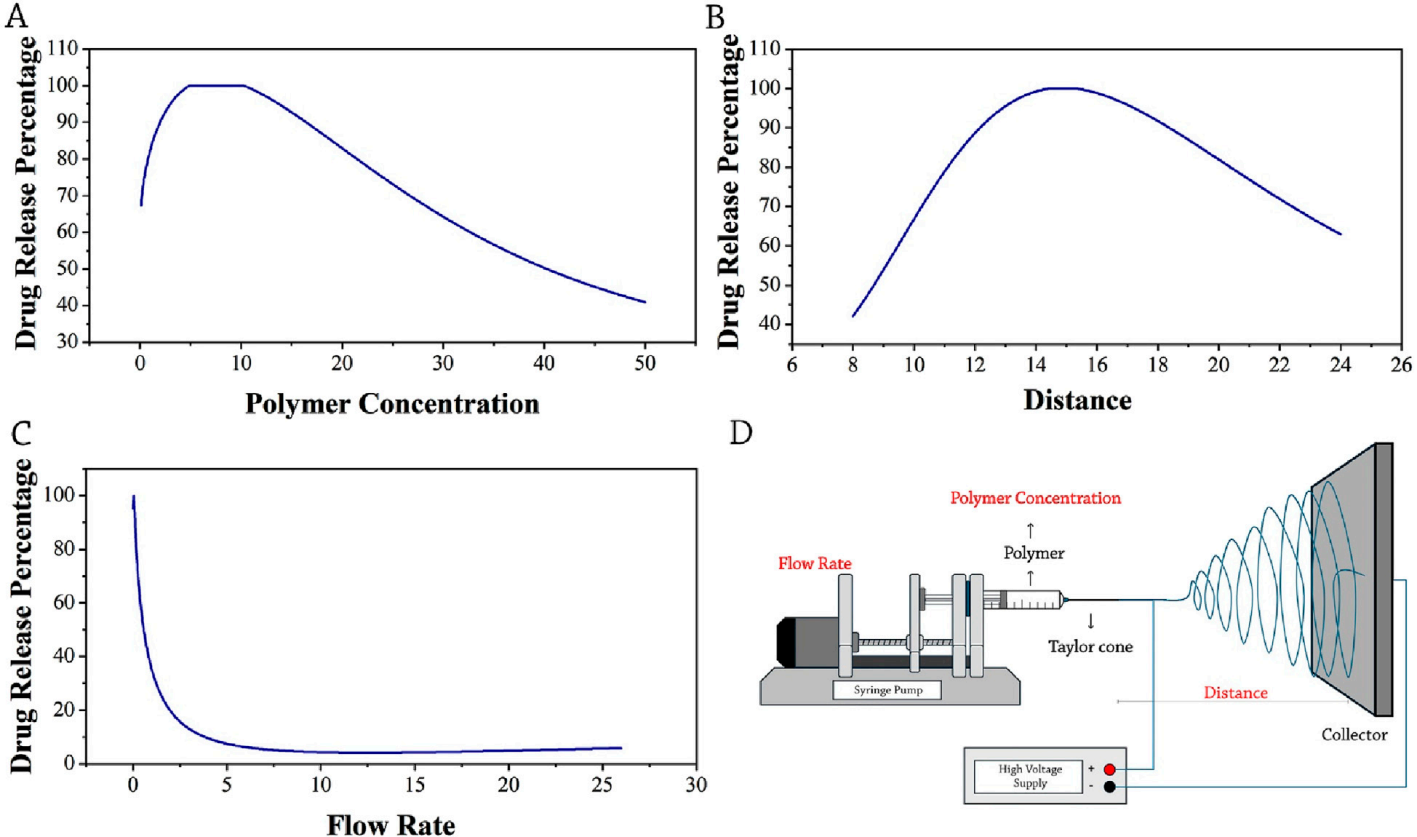
Optimization trends of drug release percentage: **(A)** polymer concentration, **(B)** distance, **(C)** flow rate, and **(D)** schematic presentation of selected parameters (red-colored) influencing the drug release percentage of drug-loaded electrospun nanofibers.

The drug release behavior within the anticipated time frame is a crucial attribute of electrospun nanofibers that significantly influences the research application and objectives (Khedri et al., 2022). According to the analysis of the data, the most important input parameters for controlling drug release are polymer concentration, the distance between the needle and collector, and flow rate. The release rate of the drug from the electrospun fibers was primarily influenced by the concentration of the polymer (Figure 2C). A greater polymer concentration results in a higher drug release over time. An upward trend is observed in the amount of release as the polymer concentration ranges from 1 to 11, reaching a maximum release of approximately 100% at a polymer concentration in the range of 6.618%–11% (Figure 5A).

As the concentration increases, we observe enhanced stability and a corresponding reduction in drug release at higher concentrations. Data analysis reveals that the optimal threshold for drug release is a polymer concentration of 6.618%–11%. The presence of high concentrations likely hindered the reduction of fiber production, as the density of the polymer posed a potential barrier to the release of drugs.

Furthermore, the distance between the needle and the collector emerges as a significant parameter in drug release. Typically, the distance falls within the range of 5–25 cm, which plays a significant role in the manufacturing process of fibers as well as their production capacity (Figure 5B). It is evident that there is a discernible upward trend in the amount of drug release when the distance is increased from 8 cm to 15 cm. Subsequently, the amount of drug release decreases. In order to have the maximum drug release percentage, the optimal point for distance between the needle and the collector is identified as 14.109 cm. Moreover, it reveals that the highest level of encapsulation has been observed at the specific coordinate of 13.073 cm. Note that the fibers exhibiting the smallest diameter have been generated at a distance of 15 cm. Thus, we can conclude that the most effective region for maximum drug release, minimum fiber diameter, and maximum encapsulation efficacy is observed within the range of 13–15 cm.

The flow rate is an additional effective parameter in the process of electrospinning. The relative impact factor of this parameter on the drug release percentage is calculated at around 70% (Figure 2C). Depending on the type of polymer solution, the flow rate in electrospinning machines is usually selected between 0.1 and 5 mL/h. However, research findings indicate that the typical flow rate used for electrospinning ranges from 1 to 3 mL/h. Furthermore, the studies revealed that a flow rate of approximately 1 mL/h yielded the maximum drug release, which significantly decreased as the flow rate escalated to 5 mL/h (Figure 5C). Dai et al. (2017) used a pearl powder/Polylactic acid (PLA) composite containing DOX for targeted drug delivery. Their results showed the optimal loading of DOX in nanofibrous scaffolds. Most importantly, the flow rate of 0.9 mL/h resulted in DOX delivery rates ranging from 59% to 97%, which increased further with the increase of pearl powder, and the anticancer effect of the drug on HeLa cells was better demonstrated (Dai et al., 2017). A schematic presentation of selected parameters (red-colored) influencing the drug release percentage is shown in Figure 5D.

### 3.7 Anticancer activity

The predominant applications of nanofibers are associated with the encapsulation and delivery of pharmaceutical substances. The effectiveness of drugs, such as those with anticancer properties, is dependent on production characteristics, encapsulation efficiency, and drug release rate. All input parameters that influence the anticancer property exhibit a relative impact factor of more than 40% (Figure 2D). The polymer density exhibited the greatest influence, which subsequently, the concentration of the polymer solution is followed by the DOX concentration, with coefficients of 80% and 60%, respectively.

**FIGURE 6:**
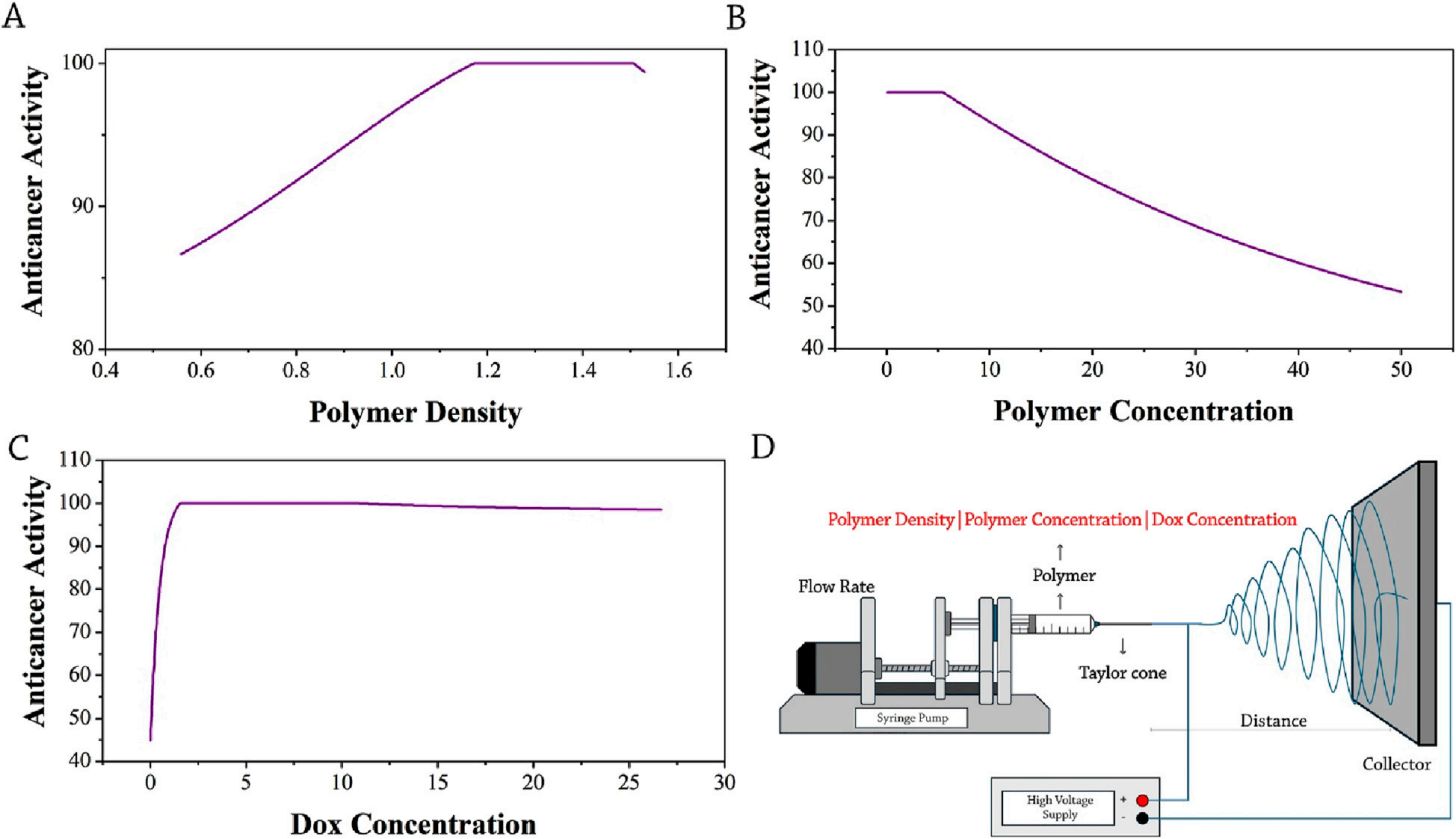
Optimization trends of anticancer activity: **(A)** polymer density, **(B)** polymer concentration, **(C)** Dox concentration, and **(D)** A schematic presentation of selected parameters (red-colored) influencing the anticancer activity of drug-loaded electrospun nanofibers.

By elevating the polymer density beyond 0.5, the activity of the anticancer properties showed a significant increase of over 80%, as presented in Figure 6A. The increased quantity has resulted in a consistent improvement in its anticancer efficacy. In the case where the polymer density value is 1.2, the anticancer property has achieved a 100% level of activity. It has been determined that a polymer density of 1.520 points is the maximum value of polymer density for the manufacturing of nanofibers, which have near 99% anticancer activity.

The concentration of the polymer is the second parameter influencing the anticancer activity of drug-loaded nanofibers, with concentrations ranging from 1 to 10 wt.% exhibiting the highest proportion of anticancer activity, followed by a decline beyond this concentration range, demonstrating the destructive effect of elevated polymer concentrations for fiber fabrication (Figure 6B).

It is essential to note that there exists a generally linear correlation between these two parameters, where an increase in polymer concentration correlates with a rise in density. Moreover, the molecular weight of the polymer significantly influences this relationship, as higher molecular weight polymers tend to form more physical entanglements, resulting in a denser material structure that can enhance the mechanical properties of the resulting fibers. These entanglements also play a crucial role in fiber formation, impacting key characteristics such as diameter, shape, and overall morphology. Higher concentrations facilitate increased entanglements, leading to better-defined fiber structures. Therefore, selecting appropriate values for both density and concentration is critical for accurate analysis, and employing empirical data alongside modeling approaches can provide deeper insights into their interactions, ultimately contributing to improved fiber quality and performance.

The drug concentration parameter is the third factor that influences the efficacy of anticancer fibers. The concentration of drugs and their subsequent release play a crucial role in determining the efficacy of the anticancer properties of nanofibers. The amount of drug released from the system directly influences the manifestation of its anticancer effects. The analysis reveals that the drug exhibits the most pronounced anticancer properties when administered at concentrations ranging from 0 to 4 wt.%, with an effective point of 3.182, representing the most pronounced impact on anticancer activity. At this particular dosage, optimal efficiency and maximum release have been attained, and higher dosages yield no further benefits (Figure 6C). A schematic presentation of selected parameters (red-colored) influencing the anticancer activity is shown in Figure 6D.

Our analysis revealed that each input (feature) could influence the outputs (labels) of the electrospinning procedure of DOX-encapsulated nanofibers. This SVM model exhibited the best optimal range of multiple features for obtaining the minimum fiber average diameter, the maximum encapsulation efficiency, the maximum drug release percentage, and the maximum anticancer activity as four labels (Table 7). The essential point to declare is that this analysis is based on the published *in vitro* and *in vivo* studies, which reported DOX-loaded nanofibers produced via an electrospinning procedure. As the anticancer study and drug release times are different in various studies, they could not have an optimum range. The overall statistics of the utilized dataset are summarized in Table 8 and extracted-data references of DOX-loaded electrospun nanofibers are gathered in Supplementary Table S1.

**TABLE 7:** The best optimal range of features for obtaining the minimum fiber average diameter, the maximum encapsulation efficiency, the maximum drug release percentage, and the maximum anticancer activity.

Features	Optimal range
Polymer Molecular Weight (Mw, kDa)	80.85–474.80
Polymer Density (g/cm3)	1.19–1.53
Additive Molecular Weight (Mw, kDa)	83.36–250.71
Additive Concentration (wt%)	0.08–23.23
Dox Concentration (wt%)	1.01–9.61
Dielectric Constant of Solvent	5.39–65.11
Polymer Concentration (wt%)	0.14–7.19
Flow Rate (mL/h)	0.81–19.65
Distance (cm)	13.07–19.54
Voltage (kV)	19.97–46.14

**TABLE 8:** The overall statistics of the utilized dataset.

	Polymer molecular weight (mw)	Polymer density (g/cm^3^)	Additive molecular weight (mw)	Additive concentration (wt%)	Dox concentration (wt%)	Dielectric constant of solvent	Polymer concentration (wt.%)	Flow rate (mL/h)	Distance (cm)	Voltage (kV)	Anticancer activity study time (day)	Anticancer activity	Average diameter (nm)	Drug release percentage (%)	Encapsulation efficiency (%)
Average	187,981.5559	1.1758	2,747.4751	5.6014	1.9406	24.7389	9.6783	1.3542	14.5734	20.3382	3.1701	36.0725	520.0117	34.2024	34.7373
Standard deviation	271433.9415	0.2273	12638.2303	6.9883	4.5333	21.1166	8.2031	3.1394	2.4776	11.7545	4.9041	24.5079	309.9338	29.7929	42.5461
Minimum	1750.36	0.559	12.01	0	0	4.81	0.1	0.006	8	0	0.02	0.07	37	0.1	0.031
1st quartile	81,000	1.117	157.24	1	0.0387	8.55	6	0.5	13.21	15	1	16.325	267	0.7775	0.7327
2nd quartile	119,250	1.19	234.4	4.1151	0.2	16.7	8	0.6	15	20	2	35.25	452.34	29	2.8
3rd quartile	172,000	1.26	853.9	5.4762	1.8772	31.3275	10	1	15	21.33	3	54.75	750	60	92
Maximum	1,300,000	1.53	100,000	30	26.66	80.1	50	26	24	95.4	35	90	1,500	99	99

## 4 Conclusion

Several techniques have been employed for the delivery of doxorubicin as a chemotherapeutic agent. Among these techniques, electrospinning has emerged as a prominent method for the fabrication of nanomaterials, owing to its favorable attributes for drug encapsulation. This approach has garnered significant attention from researchers, who have repeatedly utilized it in their investigations. These nanomaterials stand out for their remarkable drug encapsulation efficiency, enhanced by the use of cost-effective and suitable polymeric materials that can accommodate various polymer types.

Numerous investigations have been conducted to explore the encapsulation of doxorubicin within various polymeric materials, with the aim of facilitating drug delivery in cancer chemotherapy. Several parameters have been identified as influential factors in the production process, fiber characteristics, and drug loading of nanofiber materials, aiming to achieve high encapsulation efficiency and optimal anticancer activity. The aforementioned parameters are associated with the characteristics of the device as well as the properties of the polymer materials. Researchers have employed machine learning to enhance their prospective endeavors.

Our study highlights that various pertinent parameters play a significant role in the production of fibers and drug encapsulation, each possessing distinct effectiveness factors. We have demonstrated the impact of each parameter on key outcomes such as the overall features, average diameter of fibers, encapsulation efficiency, drug release, and anticancer activity. Furthermore, the optimal values for each parameter have been identified. According to our model, the distance, polymer density, and polymer concentration are the three main factors affecting the diameter of nanofibers; the distance of 15.0380 cm, the polymer density of 1.53 g/cm^3^, and polymer concentrations of below 9 wt.% were introduced as optimal values to fabricate DOX-loaded electrospun nanofibers with the minimum average fiber diameter.

The polymer concentration, dielectric constant of solvent, and machine voltage are effective parameters for encapsulation efficiency. According to our model, to have the highest encapsulation efficiency, the polymer concentration, dielectric constant of solvent, and machine voltage should be less than 15 wt.%, more than 30, and more than 20 kV, respectively. On the other hand, to have the highest release percentage, the polymer concentration should be at least 6.618 wt.%, the distance should be at least 14.109 cm, and the flow rate should be less than 5 mL/h. Optimum conditions of anticancer activity are achieved in a polymer density of 1.2–1.520 g/cm^3^, polymer concentration from 1% to 10 wt%, and Dox concentration of more than 3.182 wt.%. Therefore, to achieve optimal conditions, these values should be considered.

In this study, we clearly confirmed that the SVM machine learning model is capable of determining optimal intervals for fiber production, thereby eliminating the necessity for extensive trial and error in subsequent research endeavors. The findings demonstrate the optimal and appropriate values for each parameter, as well as the impact of each parameter on the output data. However, this model’s limitations, such as the need for significant memory to store support vectors, the long time to train large datasets, its sensitivity to noisy data and outliers, its sensitivity to kernel selection, the need for special scaling, and its difficult interpretability, have limited its application, which requires more extensive studies.

## Data Availability

The original contributions presented in the study are included in the article/Supplementary Material, further inquiries can be directed to the corresponding authors.

## Abbreviations

AI, Artificial intelligence; CNTs, Carbon nanotubes; DOX, Doxorubicin; LSSVR, Least square support vector regression model; LW-KPLSR, Locally weighted kernel partial least squares regression; ML, Machine learning; MAE, Mean Absolute Error; MSE, Mean Squared Error; PLSR, Partial least square regression; PSO, Particle Swarm Optimization; PLLA, Poly-L-lactic acid; PAN, Polyacrylonitrile; PLA, Polylactic acid; PVDF, Polyvinylidene fluoride; PCR, Principal component regression; RF, Random forest; RMSE, Root Mean Squared Error; SHCC, Secondary hepatocellular carcinoma; SVM, Support Vector Machine.
