# Supplementary material for: A supervised machine-learning analysis of doxorubicin-loaded electrospun nanofibers and their anticancer activity capabilities
Source: Front Bioeng Biotechnol. 2025 Mar 11;13:1493194. doi: 10.3389/fbioe.2025.1493194 (PMC11933076; doi:10.3389/fbioe.2025.1493194)
Supplement: Supplementary file 1 [file DataSheet1.docx]

A supervised machine-learning analysis of doxorubicin-loaded electrospun nanofibers and their anticancer activity capabilities

Mohammadreza Rostami^1, 2£^, Maliheh Gharibshahian^3, 4£^, Mehrnaz Mostafavi^5^, Ali Sufali^6^, Mahsa Golmohammadi^7^, Mohammad Reza Barati^8^, Reza Maleki^9**^, Nima Beheshtizadeh^10, 11*^

*1. Department of Nutrition, School of Allied Medical Sciences, Ahvaz Jundishapur University of Medical Sciences, Ahvaz, Iran*

*2. Food Science and Nutrition group (FSAN), Universal Scientific Education and Research Network (USERN), Tehran, Iran*

*3. Department of Tissue Engineering and Applied Cell Sciences, School of Medicine, Semnan University of Medical Sciences, Semnan, Iran*

*4. Nervous System Stem Cells Research Center, Semnan University of Medical Sciences, Semnan, Iran*

*5. Faculty of Allied Medicine, Shahid Beheshti University of Medical Sciences, Tehran, Iran*

*6. Computational Biology and Chemistry Group (CBCG), Universal Scientific Education and Research Network (USERN), Tehran, Iran*

*7. Department of Polymer Engineering and Color Technology, Amirkabir University of Technology, Tehran, Iran*

*8. Department of Advanced Materials and New Technologies, Iranian Research Organization for Science and Technology (IROST), P.O. Box 33535111, Tehran, Iran*

*9. Department of Chemical Technologies, Iranian Research Organization for Science and Technology (IROST), P.O. Box 33535111, Tehran, Iran*

*10. Department of Tissue Engineering, Faculty of Advanced Medical Sciences, Tabriz University of Medical Sciences, Tabriz, Iran*

*11. Regenerative Medicine group (REMED), Universal Scientific Education and Research Network (USERN), Tehran, Iran*

£ These two authors are first authors in this work.

Corresponding authors

*** Dr. Nima Beheshtizadeh**

Mailing address: Department of Tissue Engineering, Faculty of Advanced Medical Sciences, Tabriz University of Medical Sciences, Tabriz, Iran

Tel: +989142307282

Email: [n.beheshtizadeh@tbzmed.ac.ir](mailto:n.beheshtizadeh@tbzmed.ac.ir)

**** Dr. Reza Maleki**

Mailing address: Department of Chemical Technologies, Iranian Research Organization for Science and Technology (IROST), P.O. Box 33535111, Tehran, Iran

Tel: + 989175379884

Email: ‏


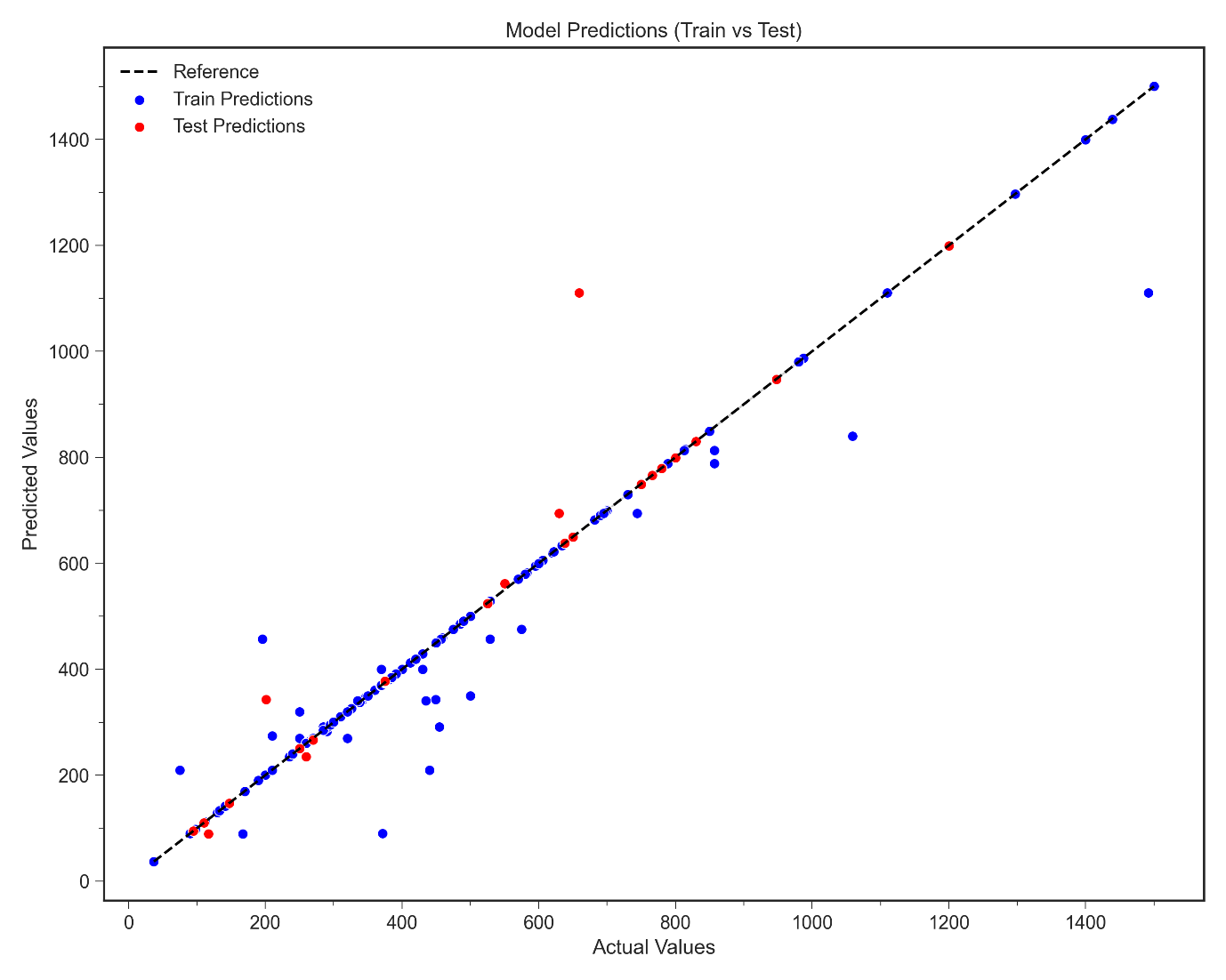


**Figure S1**. Scatter-plots for predicting average diameter


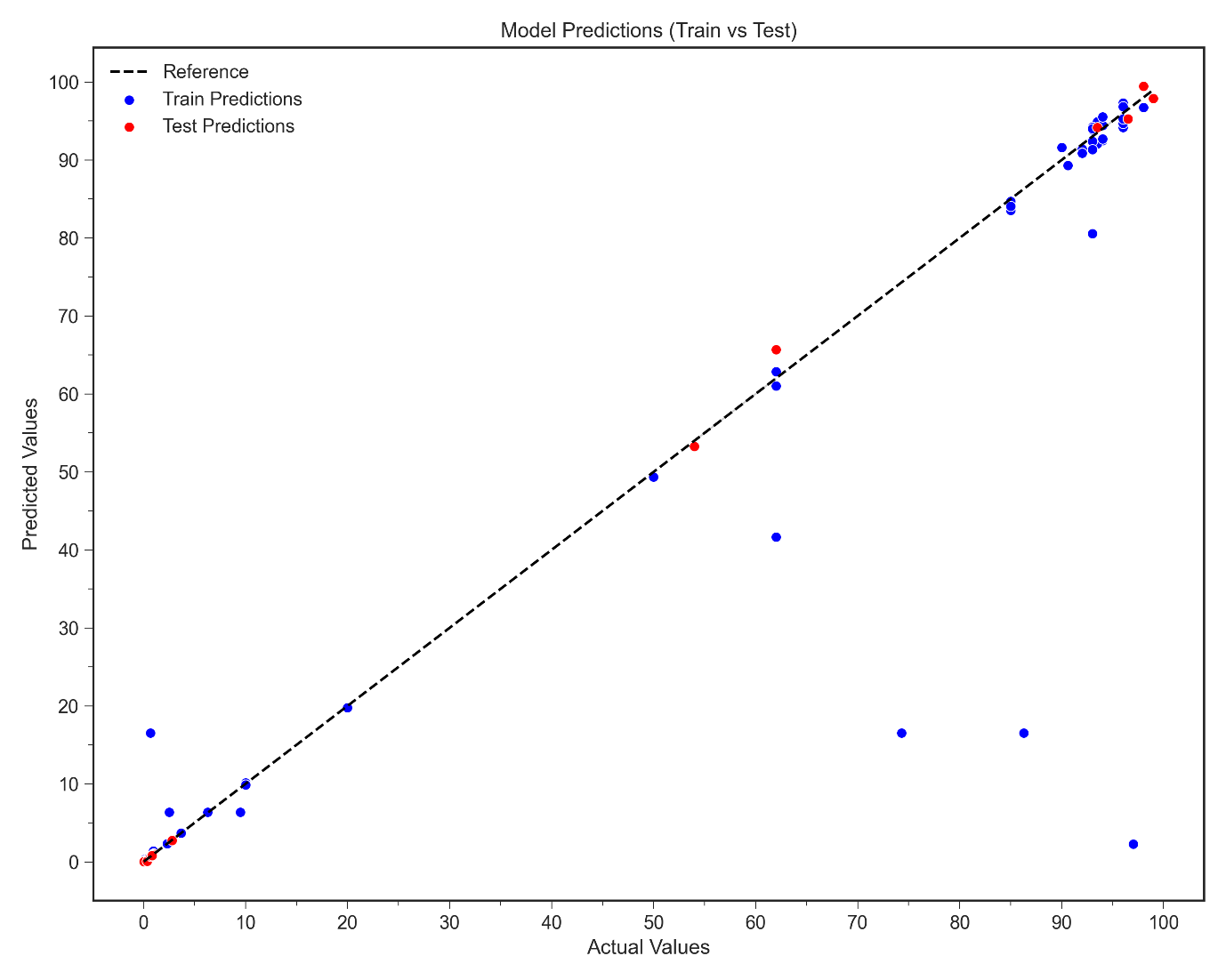


**Figure S2**. Scatter-plots for predicting encapsulation efficiency


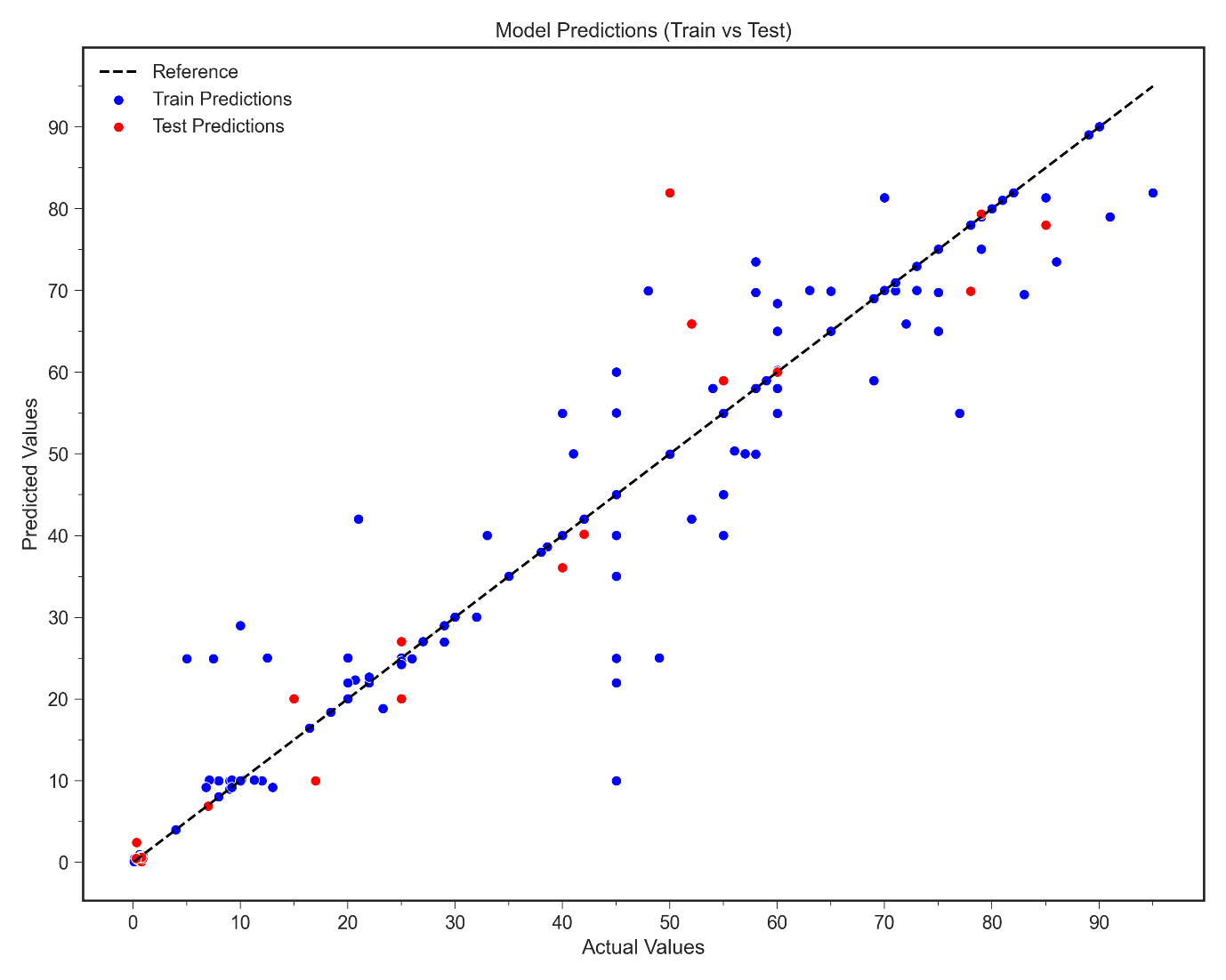


**Figure S3**. Scatter-plots for predicting drug release percentage


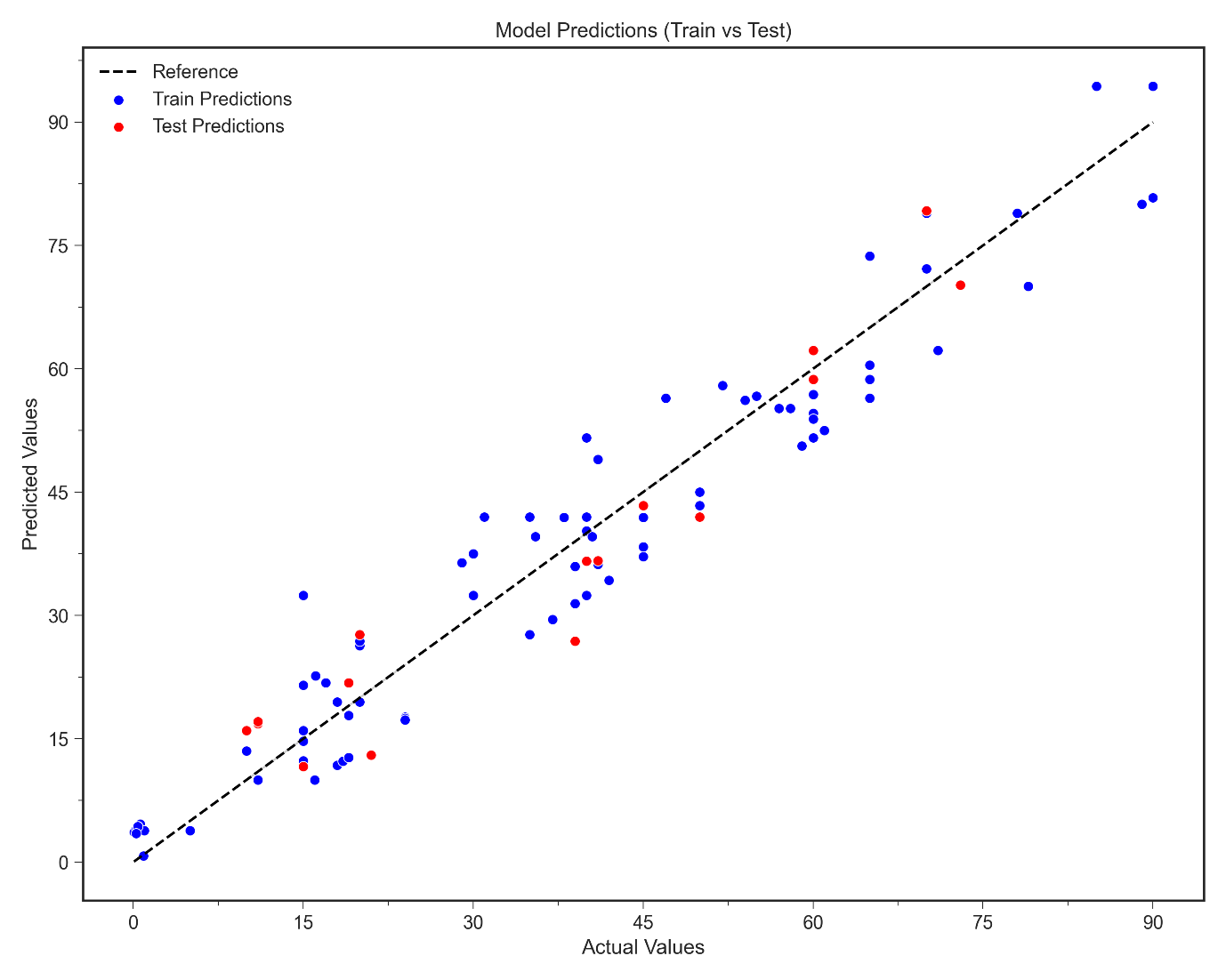


**Figure S4**. Scatter-plots for predicting anticancer activity

### **Table S1**. Extracted-data references of Doxorubicin-loaded electrospun nanofibers

| No. | Ref. |
| --- | --- |
| 1 | https://pubs.rsc.org/en/content/articlelanding/2013/tb/c3tb20636j/unauth |
| 2 | https://pubs.rsc.org/en/content/articlelanding/2013/py/c2py20779f/unauth |
| 3 | https://pubs.rsc.org/en/content/articlehtml/2013/tb/c2tb00121g |
| 4 | https://link.springer.com/article/10.1186/s11671-015-1044-7 |
| 5 | https://iopscience.iop.org/article/10.1088/0957-4484/27/24/245101/meta |
| 6 | https://www.sciencedirect.com/science/article/abs/pii/S2095927317305996 |
| 7 | https://iopscience.iop.org/article/10.1088/2053-1591/aa7479/meta |
| 8 | https://www.sciencedirect.com/science/article/pii/S1773224721002562 |
| 9 | https://link.springer.com/article/10.1007/s12668-017-0421-3 |
| 10 | https://pubs.rsc.org/en/content/articlelanding/2014/ra/c4ra09122a/unauth |
| 11 | https://journals.sagepub.com/doi/abs/10.1177/1528083716634033 |
| 12 | https://iopscience.iop.org/article/10.1088/2043-6254/aab999/meta |
| 13 | https://www.sciencedirect.com/science/article/pii/S0142961216301582 |
| 14 | https://onlinelibrary.wiley.com/doi/abs/10.1002/jbm.a.37081 |
| 15 | https://www.sciencedirect.com/science/article/pii/S0141813018302319 |
| 16 | https://www.sciencedirect.com/science/article/pii/S0141813018337917 |
| 17 | https://www.sciencedirect.com/science/article/pii/S0141813019381929 |
| 18 | https://pubs.acs.org/doi/abs/10.1021/nn504573u |
| 19 | https://www.sciencedirect.com/science/article/pii/S0939641108001203 |
| 20 | https://www.sciencedirect.com/science/article/abs/pii/S1742706113005795 |
| 21 | https://www.sciencedirect.com/science/article/abs/pii/S1567173919302019 |
| 22 | https://www.sciencedirect.com/science/article/pii/S0144861721000199 |
| 23 | https://link.springer.com/article/10.1007/s10118-016-1827-z |
| 24 | https://www.mdpi.com/1422-0067/22/5/2542 |
| 25 | https://link.springer.com/article/10.1007/s12221-020-9809-8 |
| 26 | https://onlinelibrary.wiley.com/doi/abs/10.1002/pi.6270 |
| 27 | https://www.sciencedirect.com/science/article/abs/pii/S0144861716300406 |
| 28 | https://www.sciencedirect.com/science/article/abs/pii/S0928493114008376 |
| 29 | https://www.tandfonline.com/doi/abs/10.1080/10667857.2019.1674477 |
| 30 | https://pubs.rsc.org/en/content/articlelanding/2020/tc/d0tc02604b/unauth |
| 31 | https://www.sciencedirect.com/science/article/pii/S0024320518303497 |
| 32 | https://www.sciencedirect.com/science/article/pii/S014181301732161X |
| 33 | https://aiche.onlinelibrary.wiley.com/doi/abs/10.1002/btpr.2598 |
| 34 | https://www.tandfonline.com/doi/full/10.1080/10717544.2019.1676842 |
| 35 | https://pubs.acs.org/doi/abs/10.1021/acsabm.1c00297 |
| 36 | https://www.tandfonline.com/doi/abs/10.1080/00914037.2013.854234 |
| 37 | https://www.sciencedirect.com/science/article/pii/S0939641108004281 |
| 38 | https://www.ncbi.nlm.nih.gov/pmc/articles/PMC4203157/ |
| 39 | https://www.sciencedirect.com/science/article/pii/S2468519421001294 |
| 40 | https://www.sciencedirect.com/science/article/pii/S0927776517304113 |
| 41 | http://www.nanomedicine-rj.com/article_29596_0.html |
| 42 | https://www.tandfonline.com/doi/abs/10.1080/00405000.2017.1335369 |
| 43 | https://journals.sagepub.com/doi/abs/10.1177/0883911511424655 |
| 44 | https://www.sciencedirect.com/science/article/pii/S0928493117332666 |
| 45 | https://www.sciencedirect.com/science/article/pii/S0927776517307506 |
| 46 | https://www.futuremedicine.com/doi/abs/10.2217/17435889.2.2.219 |
| 47 | https://www.tandfonline.com/doi/abs/10.1080/09205063.2015.1100495 |
| 48 | https://www.sciencedirect.com/science/article/pii/S0928098717305043 |
| 49 | https://www.sciencedirect.com/science/article/pii/S092849311832527X |
| 50 | https://www.sciencedirect.com/science/article/abs/pii/S0021979715303180 |
| 51 | https://www.cjter.com/EN/abstract/abstract3938.shtml |
| 52 | https://www.sciencedirect.com/science/article/abs/pii/S0021979721013813 |
| 53 | https://link.springer.com/article/10.1007/s00604-021-04950-7 |
| 54 | https://academic.oup.com/rb/article/8/5/rbab043/6332018?login=true |
| 55 | https://link.springer.com/article/10.1007/s00289-021-03783-x |
| 56 | https://www.sciencedirect.com/science/article/abs/pii/S0167577X15310697 |
| 57 | https://onlinelibrary.wiley.com/doi/full/10.1002/app.34463 |
| 58 | https://pubs.rsc.org/en/content/articlelanding/2013/nr/c3nr02665e/unauth |
| 59 | https://www.ncbi.nlm.nih.gov/pmc/articles/PMC6551591/ |
| 60 | https://www.sciencedirect.com/science/article/abs/pii/S1742706115300477 |
| 61 | https://www.sciencedirect.com/science/article/abs/pii/S0924224421005665 |
| 62 | https://www.mdpi.com/1996-1944/11/5/681 |
| 63 | https://www.sciencedirect.com/science/article/pii/S0168365916302747 |
| 64 | https://www.tandfonline.com/doi/abs/10.1517/17425247.2013.758103 |
| 65 | https://www.sciencedirect.com/science/article/pii/S0939641112002834 |
| 66 | https://pubs.acs.org/doi/abs/10.1021/acsabm.8b00735 |
| 67 | https://www.tandfonline.com/doi/full/10.1080/15685551.2013.771303 |
| 68 | https://en.cnki.com.cn/Article_en/CJFDTotal-JZYX201506002.htm |
| 69 | https://www.tandfonline.com/doi/abs/10.1080/09205063.2016.1203218 |
| 70 | https://pubag.nal.usda.gov/catalog/5703632 |
| 71 | https://www.sciencedirect.com/science/article/pii/S0141813018337917 |
| 72 | https://www.sciencedirect.com/science/article/abs/pii/S0927776515002015 |
| 73 | https://www.sciencedirect.com/science/article/pii/S0927776519302498 |
| 74 | https://pubs.acs.org/doi/abs/10.1021/acsami.6b04424 |
| 75 | https://pubs.rsc.org/en/content/articlehtml/2017/ra/c7ra12394a |
| 76 | https://www.sciencedirect.com/science/article/abs/pii/S0928493115304069 |
| 77 | https://link.springer.com/article/10.1007/s11095-018-2442-1 |
| 78 | https://onlinelibrary.wiley.com/doi/full/10.1002/mabi.201300575 |
| 79 | https://www.sciencedirect.com/science/article/abs/pii/S0928493114002458 |
| 80 | https://www.sciencedirect.com/science/article/abs/pii/S002197971400839X |
| 81 | https://onlinelibrary.wiley.com/doi/abs/10.1002/app.50041 |
| 82 | https://pubs.rsc.org/en/content/articlehtml/2014/ra/c4ra03722g |
| 83 | https://www.sciencedirect.com/science/article/pii/S0168365917305904 |
| 84 | https://www.sciencedirect.com/science/article/pii/S0928493119343930 |
| 85 | https://www.sciencedirect.com/science/article/pii/S1773224719309852 |
| 86 | https://onlinelibrary.wiley.com/doi/abs/10.1002/pola.29422 |
| 87 | https://pubs.rsc.org/en/content/articlehtml/2015/ra/c5ra11830a |
| 88 | https://onlinelibrary.wiley.com/doi/abs/10.1002/adhm.201701024 |
| 89 | https://onlinelibrary.wiley.com/doi/full/10.1002/mame.201500160 |
| 90 | https://www.sciencedirect.com/science/article/abs/pii/S0928493112004468 |
| 91 | https://www.tandfonline.com/doi/abs/10.1080/03639045.2020.1730397 |
| 92 | https://link.springer.com/article/10.1007/s42765-019-00007-w |
| 93 | https://www.sciencedirect.com/science/article/pii/S0928493116319889 |
| 94 | https://pubs.rsc.org/en/content/articlelanding/2018/bm/c7bm01018d/unauth |
| 95 | https://pubs.acs.org/doi/abs/10.1021/acsbiomaterials.6b00046 |
| 96 | https://link.springer.com/article/10.1007/s43440-021-00220-8 |
| 97 | https://www.sciencedirect.com/science/article/abs/pii/S1385894720313942 |
| 98 | https://pubs.acs.org/doi/abs/10.1021/acsnano.8b01729 |
| 99 | https://www.sciencedirect.com/science/article/pii/S0144861721000679 |
| 100 | https://onlinelibrary.wiley.com/doi/full/10.1002/app.41286 |
| 101 | https://onlinelibrary.wiley.com/doi/abs/10.1002/smll.201801183 |
| 102 | https://pubs.rsc.org/en/content/articlelanding/2021/xx/c9bm00756c/unauth |
| 103 | https://www.sciencedirect.com/science/article/pii/S0141813016327489 |
| 104 | https://www.sciencedirect.com/science/article/pii/S0142961215009655 |
| 105 | https://link.springer.com/article/10.1007/s10570-020-03459-1 |
| 106 | https://www.mdpi.com/2079-4991/9/4/656 |
| 107 | https://hal.archives-ouvertes.fr/hal-02192566/ |
| 108 | https://www.sciencedirect.com/science/article/pii/S0928493120333026 |
| 109 | https://www.sciencedirect.com/science/article/pii/S0378517320303963 |
| 110 | https://www.sciencedirect.com/science/article/abs/pii/S0167577X15310697 |
| 111 | https://www.sciencedirect.com/science/article/pii/S0272884217303930 |
| 112 | https://pubs.acs.org/doi/abs/10.1021/acs.langmuir.6b02227 |
| 113 | https://onlinelibrary.wiley.com/doi/abs/10.1002/marc.201800058 |
| 114 | https://pubs.acs.org/doi/abs/10.1021/acsbiomaterials.9b01313 |
| 115 | https://pubs.acs.org/doi/abs/10.1021/acs.jafc.9b06588 |
| 116 | https://onlinelibrary.wiley.com/doi/abs/10.1002/marc.201900499 |
| 117 | https://www.tandfonline.com/doi/abs/10.1080/03602532.2019.1642912 |
| 118 | https://www.sciencedirect.com/science/article/pii/S0927776520302356 |
| 119 | https://www.sciencedirect.com/science/article/abs/pii/S1385894718309264 |
| 120 | https://www.sciencedirect.com/science/article/abs/pii/S0753332216329304 |
| 121 | https://www.sciencedirect.com/science/article/pii/S0168365905003500 |
| 122 | https://pubs.acs.org/doi/abs/10.1021/la402080y |
| 123 | https://pubs.rsc.org/en/content/articlelanding/2016/bm/c6bm00070c/unauth |
| 124 | https://link.springer.com/article/10.1007/s42765-020-00053-9 |
| 125 | https://pubs.rsc.org/en/content/articlelanding/2015/tb/c5tb00206k/unauth |
| 126 | https://onlinelibrary.wiley.com/doi/abs/10.1002/mame.202000457 |
| 127 | https://www.sciencedirect.com/science/article/pii/S0168365920306866 |
| 128 | https://link.springer.com/article/10.1007/s11095-006-9036-z |
| 129 | https://pubs.rsc.org/en/content/articlelanding/2021/nj/d1nj02159a/unauth |
| 130 | https://onlinelibrary.wiley.com/doi/abs/10.1002/smll.201804397 |
